# Comparison of Screening Mammogram Rates Before vs During the COVID-19 Pandemic Among Medicaid Beneficiaries in Louisiana

**DOI:** 10.1001/jamanetworkopen.2022.51687

**Published:** 2023-01-19

**Authors:** Yixue Shao, Kevin Callison, Andrew Anderson, Thomas A. LaVeist, Brigham Walker

**Affiliations:** 1 Department of Health Policy and Management, Tulane University School of Public Health and Tropical Medicine, New Orleans, Louisiana

## Abstract

This cohort study investigates differences in screening mammography before vs during the COVID-19 pandemic by race and ethnicity among Medicaid beneficiaries in Louisiana.

## Introduction

The COVID-19 pandemic and related facility closures disrupted preventive care use,^1^ with screening mammography decreasing by 60% between March and July 2020.^2^ Although the pandemic was associated with disproportionate outcomes among historically marginalized populations,^3^ its association with screening mammography rates by race and ethnicity among Medicaid beneficiaries has yet to be explored. Disparities associated with the pandemic are especially relevant for Medicaid beneficiaries living in Southern states given long-standing barriers to care, relatively low screening rates, and relatively high cancer mortality rates.^4^ Using Louisiana Medicaid claims data, we examined differences in screening mammography before vs during the COVID-19 pandemic by race and ethnicity.

## Methods

This cohort study followed the STROBE reporting guideline and was exempted from review by the Institutional Review Board of Tulane University under the Common Rule, 45 CFR §46.104(d)(4)(ii). We obtained claims data for non–dual-eligible female beneficiaries aged 50 to 64 years who were continuously enrolled in Louisiana Medicaid from January 2018 through August 2021. We identified screening mammograms using Current Procedural Terminology codes (77057, 77063, and 77067) and Healthcare Common Procedure Coding System code G0202. We plotted changes in monthly screening mammogram rates and tested for statistical differences by race and ethnicity (self-report) before and during the COVID-19 pandemic using Wald tests in Stata statistical software version 17.0 (StataCorp).

## Results

The Table presents demographic characteristics for 43 926 female beneficiaries in our sample (mean [SD] age, 55.04 [4.30] years; 1198 Hispanic [2.7%], 21 615 non-Hispanic Black [49.2%], and 21 113 non-Hispanic White [48.1%]). Screening mammogram rates decreased to nearly 0 during the first wave of peak COVID-19 infections in Louisiana in April 2020 and remained approximately 30% below baseline (2018-2019) levels from May through August 2020 as measured by number of fewer monthly screens per 1000 beneficiaries (−11.0 screens; 95% CI, −17.2 to −4.8 screens) (Figure). COVID-related screening decreases from April through August 2020, as number of fewer monthly screens per 1000 beneficiaries, were similar across race and ethnicity groups (non-Hispanic Black: −17.4 screens; 95% CI, −26.8 to −8.0 screens; 54.2% decrease; Hispanic: −14.3 screens; 95% CI, −23.7 to −4.9 screens; 40.1% decrease; non-Hispanic White: −14.9 screens; 95% CI, −24.2 to −5.5 screens; 57.0% decrease).

**Table.** Demographic Characteristics

Characteristic	Beneficiaries, No. (%) (N = 43 926)^a^		
	Black (n = 21 615 [49.2%])	Hispanic (n = 1198 [2.7%])	White (n = 21 113 [48.1%])
Age, mean (SD), y	54.92 (4.34)	55.04 (4.41)	55.17 (4.24)
Geography			
Urban	15 453 (71.49)	757 (63.19)	12 860 (60.91)
Rural	6162 (28.51)	441 (36.81)	8253 (39.09)

^a^ Data source: Louisiana Medicaid claims data warehouse, January 2018 to August 2021.

**Figure.**
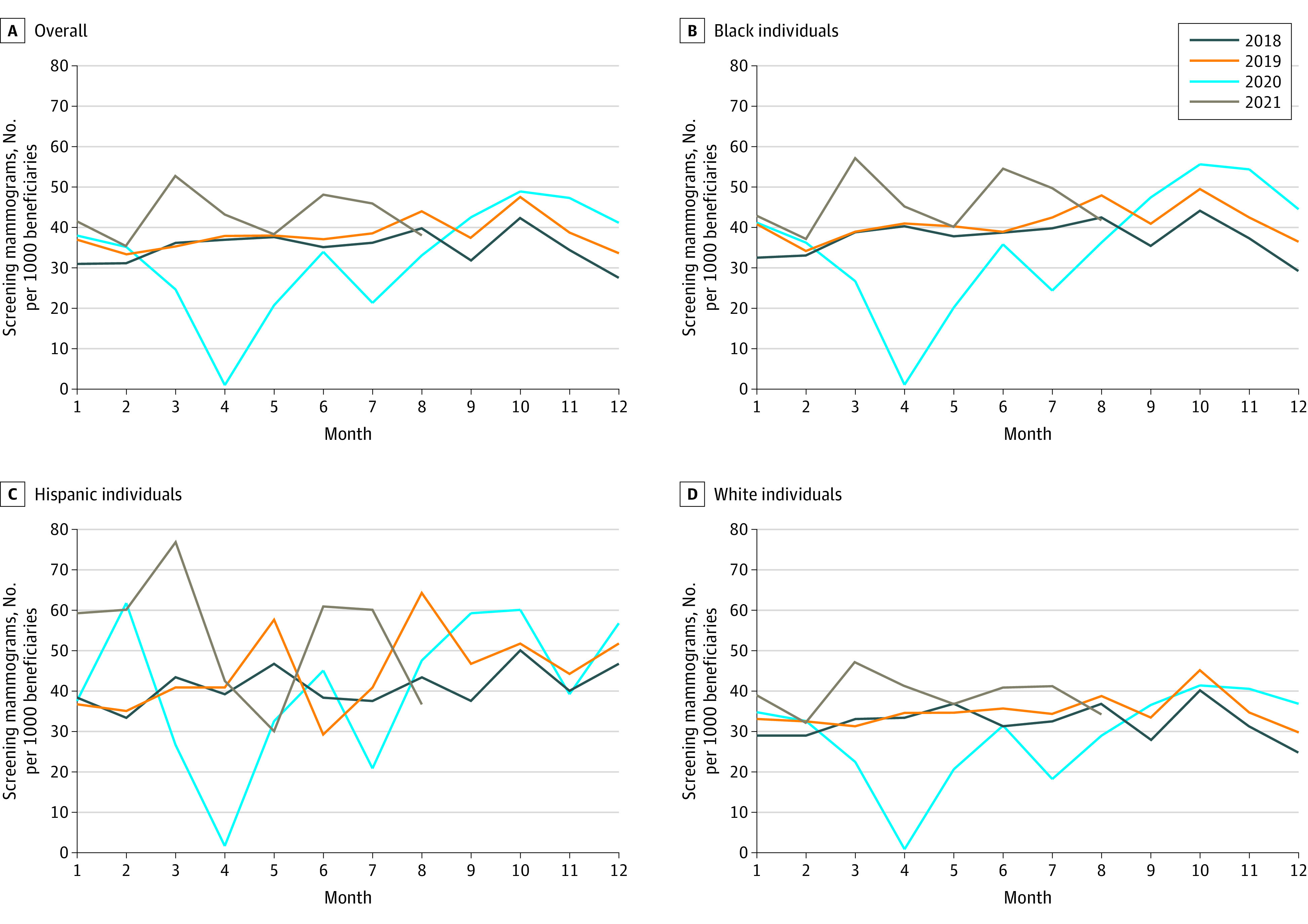
Trends in Mammogram Use January 2018 to August 2021

Screening mammography rates had rebounded by September 2020 and remained at or above baseline levels through August 2021. Non-Hispanic Black individuals had a mean of an additional 8.2 screenings (95% CI, 3.6-12.9 screenings) per 1000 beneficiaries per month compared with the same months in 2018 and 2019, a 22.2% increase. Hispanic individuals had a mean of an additional 10.4 screenings (95% CI, 5.7-15.0 screenings) per 1000 beneficiaries per month, a 25.3% increase. Non-Hispanic White individuals had a mean of an additional 5.5 screenings (95% CI, 0.9-10.2 screenings) per 1000 beneficiaries per month, a 17.6% increase. There were no significant differences in rate increases by race or ethnicity.

Overall, individuals in our sample received 3538 fewer screening mammograms (95% CI, −3760.1 to −3314.9 screenings) from April through August 2020 compared with the mean for the same months over 2018 and 2019. However, screening mammography rebounded rapidly beginning in September 2020, and by July 2021, excess screenings over 2018 to 2019 means had more than compensated for COVID-related screening decreases.

## Discussion

This cohort study found that screening mammography declined sharply for Louisiana Medicaid beneficiaries during the first 2 waves of peak COVID-19 infections. Although a recent national study found that screening mammography had not yet returned to prepandemic levels,^5^ we found that screenings among Louisiana Medicaid beneficiaries had fully recovered by mid-2021. Increases were most pronounced for non-Hispanic Black and Hispanic beneficiaries, a finding consistent with higher prepandemic screening rates for these groups in Louisiana,^6^ although differences across groups were not statistically significant.

Our analysis was limited to a single state and focused on a low-income population. However, our study may provide new evidence of the association of the pandemic with screening mammography rates. Future research should explore whether delayed breast cancer screenings among Medicaid beneficiaries were associated with later-stage diagnoses or poorer prognoses for individuals diagnosed with breast cancer.

## Notes

Supplement.
Data Sharing Statement
