# Supplementary material for: Comparison of Screening Mammogram Rates Before vs During the COVID-19 Pandemic Among Medicaid Beneficiaries in Louisiana
Source: JAMA Netw Open. 2023 Jan 19;6(1):e2251687. doi: 10.1001/jamanetworkopen.2022.51687 (PMC9857623; doi:10.1001/jamanetworkopen.2022.51687)
Supplement: Supplement. — Data Sharing Statement [file jamanetwopen-e2251687-s001.pdf]

Data Sharing Statement

Shao. Comparison of Screening Mammogram Rates Before vs During the COVID-19 Pandemic Among Medicaid Beneficiaries in Louisiana. *JAMA Netw Open*. Published January 19, 2023. doi:10.1001/jamanetworkopen.2022.51687

Data
Data available: No

Additional Information
Explanation for why data not available: The data belong to the state of Louisiana, not to the researchers (we access it through a data use agreement with the state)

© 2023 Shao Y et al. *JAMA Network Open*.
